# Magnitude, Severity, and Morphological Types of Anemia in Hospitalized Children Under the Age of Five in Southern Tanzania

**DOI:** 10.7759/cureus.1499

**Published:** 2017-07-21

**Authors:** Fabian P Mghanga, Christopher M Genge, Leonia Yeyeye, Zainab Twalib, Wilfred Kibopile, Fredrick J Rutalemba, Tito M Shengena

**Affiliations:** 1 Faculty of Medicine, Archbishop James University College; 2 Department of Obstetrics and Gynecology, Mtwara Clinical Officers Training Centre, Mtwara, Tanzania; 3 Department of Pediatrics, Mtwara Clinical Officers Training Centre, Mtwara, Tanzania; 4 Department of Internal Medicine, Mtwara Clinical Officers Training Centre, Mtwara, Tanzania

**Keywords:** anaemia, prevalence, hospitalized under-five children, tanzania

## Abstract

Introduction

Anemia is a significant public health problem among children and women globally. It is one of the most common causes of deaths among children admitted to hospitals in sub-Saharan Africa. Case fatality rates of 6 percent to 18 percent have been reported even in facilities that have blood transfusions services. The purpose of this study was to evaluate the magnitude, severity, and morphological types of anemia among hospitalized children under five years of age in the southern part of Tanzania.

Methods

A cross-sectional, hospital-based, retrospective analysis was conducted in February 2016 using hospital records of 303 children aged 0-59 months admitted to St. Benedict Ndanda Referral Hospital, Mtwara, Tanzania between 1 July 2015 and 31 December 2015.

Results

The mean hemoglobin (Hb) level of the study population was 7.87 ± 2.84 g/dL, the median was 8.00g/dL, the interquartile range (IQR) was 4.40g/dL, and the prevalence of anemia was 83.17 percent. The magnitude of mild, moderate, and severe anemia was 9.13 percent, 44.84 percent, and 46.03 percent, respectively, and about half of all anemic children had normocytic anemia.

Conclusion

Severe anemia is a common health problem among hospitalized children under five years of age in the study area. We recommend screening all admitted children under the age of five for anemia, and clinicians should pay attention to and put more emphasis on intervention strategies for anemia when treating children admitted for other diseases.

## Introduction

Anemia is a global public health problem that affects people in both developing and developed countries associated with an increased risk of morbidity and mortality, especially among pregnant women and children under five years of age [1]. It is estimated that approximately 24.80 percent of the global population is anemic [2], while an estimated 47.40 percent of preschool and 25.40 percent of schoolchildren suffer from this disease worldwide [2-3]; 28.50 percent of these children are found in sub-Saharan Africa [4].

Low hemoglobin levels in children under the age of five have been shown to be associated with factors such as lack of awareness of anemia in parents/guardians coupled with their low educational status, poor nutritional practices, unhealthy food habits, a diet with low iron bioavailability, and malaria and parasitic infestations [5-8].

The condition is a critical public health problem in most sub-Saharan African countries with the prevalence of anemia among the under-five children in the community ranging from 37.30 percent in Ethiopia [9] to 72.00 percent in Uganda [10]; in hospitalized under-five patients, the prevalence reaches up to 77.20 percent in western Tanzania [11].

Hospital records in southern Tanzania indicate that many children under five children suffer from anemia, and the condition ranks among the leading causes of mortality in pediatrics wards. It is estimated that approximately 79.60 percent [3] of the preschool population is anemic, causing deaths in 17.80 percent of hospitalized children under five years of age, ranking second after malaria [12]. Despite these records, the true magnitude of the problem is still unknown, as no studies have been conducted in this study area.

Prompted by this relatively high prevalence of anemia among children under five years of age attending health care facilities, combined with the fact that, currently, there is limited information on the prevalence and determinants of anemia among under-five children in Southern Tanzania, we conducted this hospital-based study to determine the prevalence, severity, and morphological types of anemia in hospitalized children under five years of age.

## Materials and methods

Study Design and Study Setting

A cross-sectional analysis of hospital records data collected from 1 July 2015 to 31 December 2015 at St. Benedict Ndanda Referral Hospital, Mtwara, Tanzania, was conducted between February and March 2016. St. Benedict Ndanda Referral Hospital is a 320-bed referral hospital and is among the largest hospitals in Southern Tanzania. Being a referral hospital, it has a big turnover of patients from all over the southern part of the country and neighboring Mozambique and attends an average of 10,000 (both inpatient and outpatient) children aged 6 to 59 months annually. Although it is in a rural area, the hospital was selected because of its good record keeping. It also has one of the best laboratories in the southern zone with trained personnel and modern equipment.

Study Sample and Data Collected

The demographic data and hematological laboratory parameters of all children aged 6 to 59 months admitted at St. Benedict Ndanda Referral Hospital, Tanzania between 1 July 2015 and 31 December 2015 were collected from patient files using closed-ended questionnaires. The patient's hospital records were available for 6,055 children aged 6 to 59 months, in which 3,399 children were attended on an outpatient basis and 2,656 children were inpatients. Full blood counts were available for 834 of the hospitalized patients and were included in the analysis. Hospitalized patients were recruited from the pediatric wards of the hospital. We excluded the case files of children with bleeding disorders, active hemorrhage,  human immunodeficiency virus (HIV) infected children, and children with a history of blood transfusion and/or surgery within two months prior to admission. For children who had more than one measurements done during the six-month period, we only took into account the most recent measurement of hemoglobin to avoid repeated measurements of hemoglobin in the same patient.   

**Figure 1:**
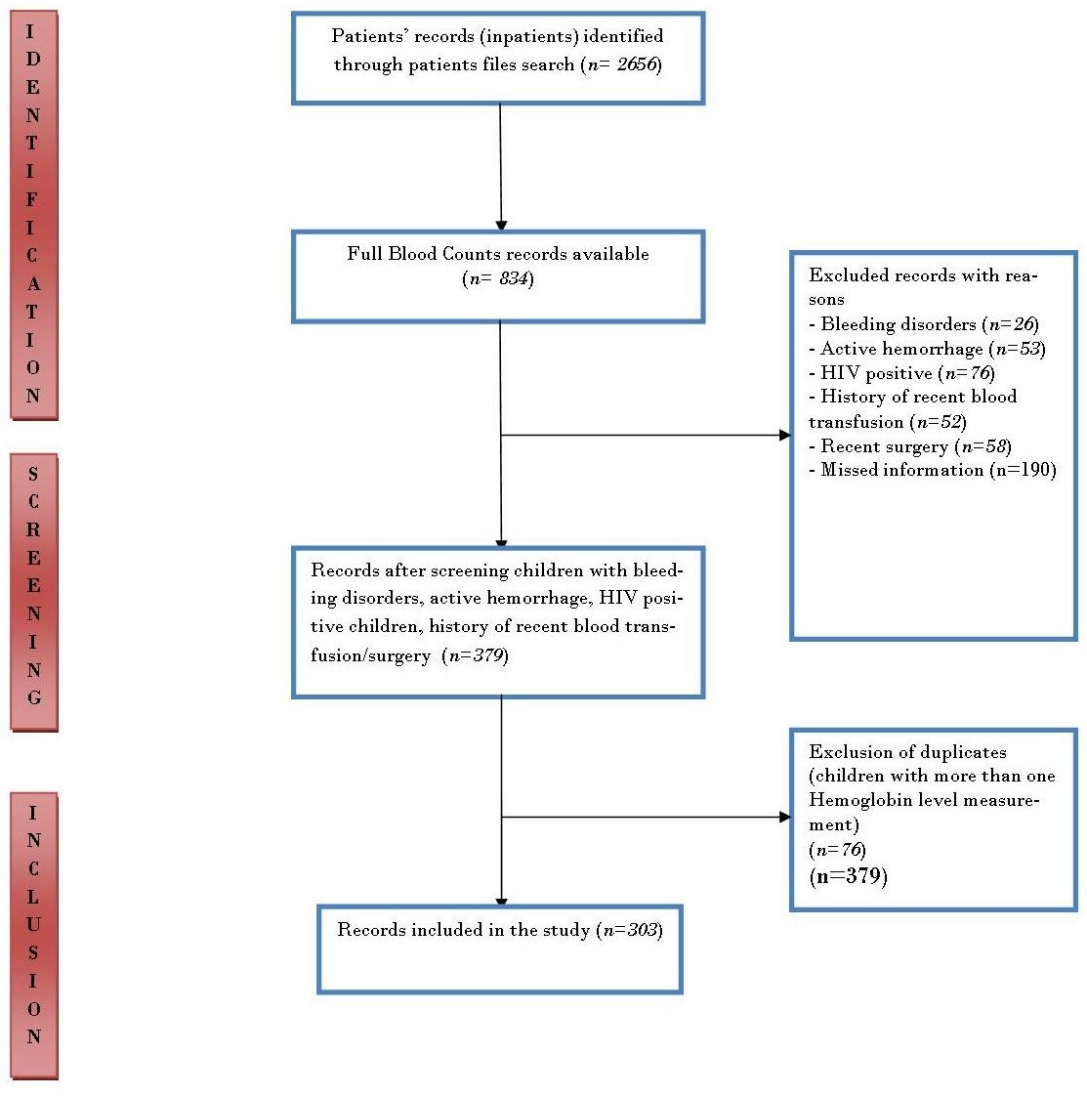
Selection process of patients included in the study

We also collected the following information: children's age, sex, residence, history of sickle cell disease (SCD) or repeated blood transfusions, history of chronic illnesses, guardian's occupation, the educational level of the parents/guardians, and physical examination findings, such as palmar and conjunctival pallor and scleral jaundice. We excluded patients without the above information.  A total of 303 children were included in the study (Figure 1).

Definitions of Variables

The independent variables studied included sex, age, residence, and working diagnosis, whereas the dependent variables were hemoglobin levels and the presence of anemia by severity and morphology. Anemia for children aged between 6 and 59 months was defined according to World Health Organization (WHO) criteria [13]. Normocytic anemia was defined as a mean corpuscular volume (MCV) between 80 and 100 fl, microcytic anemia as an MCV below 80 fl, and macrocytic anemia by an MCV above 100 fl [14]. All hemoglobin level estimations were done by the Hemo Control EKF diagnostic machine (EKF Diagnostics, Germany) while full blood pictures were analyzed using an automated hematology analyzer, CELL-DYN 3700 (Abbott Laboratories, USA).

Statistical Analysis

Data were double-entered using Microsoft Excel 2013, cleaned, and analyzed using Stata version 14 (StataCorp. 2015. College Station, Texas, USA). Missing or implausible data were clarified. For the description of continuous vari­ables, we used summary statistics (mean and standard deviation), the interquartile range (IQR), and the median, and for categorical variables, we reported the number and percentage of participants in each category. We applied univariate and multivariate logistic regression analyses to determine the predictors of anemia. We also used the chi-square test to determine the association between the mean corpuscular volume and sex and white blood cell (WBC) total counts. All statistical tests were conducted at a 0.05 level of significance.

Ethical Approval

The study was approved by the Ligula Hospital Ethical Committee. The objectives, importance, and procedures of the study were explained to the hospital management and confidentiality was assured.

## Results

Demographic Characteristics of Study Subjects

A total of 303 patient reports were included in the study. Fifty-seven percent (n = 172) of studied children were males and 43 percent (n = 131) were females. The mean age was (24.45 ± 13.45) months (median: 25.6 months, range: 6 – 58 months). Children below two years of age constituted the largest proportion (59.08 percent) of the study population. The IQR, median, and mean hemoglobin level of the study population was 4.40 g/dL, 8.00 g/dL, and 7.87 ± 2.84 g/dL, respectively.

Prevalence of Anemia

The laboratory results of the full blood counts retrieved showed that 16.83 percent (n=51) of all attended under-five children had normal hemoglobin levels and 83.17 percent (n=252) were anemic. At least 58 percent (n=146) of anemic children were boys, with less than a quarter of both boys and girls (15.12 percent and 19.08 percent, respectively) having normal hemoglobin levels. Male children had slightly lower hemoglobin mean values compared to females although the difference was not statistically significant (p=0.65). Table 1 shows the demographic characteristics of study subjects and the distribution of children with and without anemia among different groups.

**Table 1:** The distribution of anemia among various groups of hospitalized children (n=303) Note: Age: mean ± SD, months: 24.45 ± 13.45, range 6 – 58 months Abbreviations: n: positive number; N: total number evaluated; SD: standard deviation; AWD: acute watery diarrhea; UTI: urinary tract infection; SAM: severe acute malnutrition; RTIs: respiratory tract infections

Variables	Distribution		Hemoglobin Values (g/dl)	Prevalence of Anaemia			
				Non-anemic		Anemic	
	*n*	*%*	mean± SD	*n*	*%*	*n*	*%*
Total population	303/303	100.00	7.87 ± 2.84	51/303	16.83	252/303	83.17
Sex							
* Males*	172/303	56.77	7.80 ± 2.75	26/172	15.12	146/172	84.88
* Females*	131/303	43.23	7.95 ± 2.94	25/131	19.08	106/131	80.92
Age (months)							
* 6 – 24*	179/303	59.08	7.86 ± 2.88	25/179	13.97	154/179	86.03
* 25 – 59*	124/303	40.92	7.88 ± 2.78	26/124	20.97	98/124	79.03
Diagnosis at admission							
* Malaria*	174/303	57.43	7.03 ± 2.70	16/174	9.20	158/174	90.80
* RTIs*	49/303	16.17	8.62 ± 2.60	12/49	24.49	37/49	75.51
* Other diagnoses*	27/303	8.91	9.08 ± 3.51	9/27	33.33	18/27	66.67
* Septicemia*	18/303	5.94	8.81 ± 1.74	2/18	11.11	16/18	88.89
* AWD*	13/303	4.29	10.82 ± 1.54	7/13	53.85	6/13	46.15
* UTI*	7/303	2.31	8.73 ± 2.48	2/7	28.57	5/7	71.43
* SAM*	7/303	2.31	7.61 ± 2.40	1/7	14.29	6/7	85.71
* Gastroenteritis*	8/303	2.64	9.98 ± 1.24	2/8	25.00	6/8	75.00
Residence							
* Rural*	159/303	52.48	7.84 ± 2.83	27/159	16.98	132/159	83.02
* Urban*	144/303	47.52	7.89 ± 2.85	24/144	16.67	120/144	83.33

Anemia and Diagnosis at Admission

We performed a univariate logistic regression analysis to test for the effect of malaria, respiratory tract infections, septicemia, acute watery diarrhea (AWD), urinary tract infections (UTI), severe acute malnutrition (SAM), gastroenteritis, and other diagnoses on the risk of acquiring anemia, with adjustments for age and sex. The analysis revealed that respiratory tract infections (p=0.01), other diagnoses (p<0.05), and AWD (p<0.05) were associated with a higher risk of anemia (Table 2).

**Table 2:** The logistic regression analysis on diagnosis at admission and the risk of acquiring anemia Abbreviations: p: probability of Chi-square test; AWD: acute watery diarrhea; UTI: urinary tract infection; SAM: severe acute malnutrition; RTIs: respiratory tract infections

Diagnosis at admission	Odds Ratio		Standard Error	p-value	[95% Conf. Interval]
Malaria	1.00		(base)		
*RTIs*	0.31		0.13	0.01	0.14 - 0.72
*Other diagnoses*	0.20		0.10	0.00	0.08 - 0.52
*Septicemia*	0.81		0.64	0.79	0.17 - 3.84
*AWD*	0.09		0.05	0.00	0.03 - 0.29
*UTI*	0.25		0.22	0.12	0.05- 1.41
*SAM*	0.61		0.68	0.65	0.07 - 5.37
*Gastroenteritis*	0.30		0.26	0.17	0.06 - 1.63

A multivariate regression analysis was performed to evaluate the effects of age, sex, diagnosis at admission, and the residence of the hospitalized children on the risk of acquiring anemia The findings show that 4.0 percent of the variation of the anemia outcome is explained by the regressors of age, sex, diagnosis at admission, and the residence of the attended children (Table 3), while only the diagnosis at admission was statistically significantly associated with the risk of acquiring anemia (p<0.05) with age, sex, and the residence of children showing no statistical significance (p-values are 0.27, 0.33, and 0.69, respectively).

**Table 3:** Results of the multivariate logistic regression analyses for the multivariate regression model on the risk of acquiring anemia Abbreviations: OR: odds ratio; 95% CI: 95% confidence interval; p: probability of Chi-square test; AWD: acute watery diarrhea; UTI: urinary tract infection; SAM: severe acute malnutrition; RTIs: respiratory tract infections

	OR	Standard Error	95% CI	*p*
Sex				
* Males*	1.00	(base)		
* Females*	0.76	0.25	0.40 – 1.42	0.41
Age (months)				
* 6 – 24*	1.00	(base)		
* 25 – 59*	0.47	0.16	0.24 – 0.93	0.03
Diagnosis at admission				
* Malaria*	1.00	(base)		
* RTIs*	0.26	0.12	0.11 – 0.62	0.00
* Other diagnoses*	0.22	0.11	0.08 – 0.58	0.00
* Septicemia*	0.67	0.54	0.14 – 3.25	0.62
* AWD*	0.07	0.05	0.02 – 0.25	0.00
* UTI*	0.20	0.18	0.03 – 1.16	0.07
* SAM*	0.73	0.82	0.08 – 6.57	0.78
* Gastroenteritis*	0.32	0.28	0.06 – 1.79	0.20
Residence				
* Rural*	1.00	(base)		
* Urban*	1.21	0.41	0.62 – 2.36	0.57

Classification of Anemia Based on Severity

A multinomial logistic regression model was used to predict the severity of anemia among children (Table 4). The omitted comparison categories in the model were male gender with anemia (n =146), anemic children below two years (n=154), anemic children with malaria (n=158), and anemic children residing in rural areas (n=132). Predictor variables in the model included sex, age, diagnosis at admission, and residence. Severe anemia (defined as hemoglobin less than 7.00 g/dL) was found in 46.03 percent (n=116) of anemic patients. Children above two years of age (regular rate and rhythm (RRR) = 0.75, p = 0.67) had lower relative risk reduction of being severely anemic, while children diagnosed with AWD (p=0.99) and gastroenteritis (p = 0.99) had very high RRR, respectively. Moderate anemia accounted for 44.84 percent (n = 113) of all anemic children. Girls (RRR = 0.57, p=0.28) and children diagnosed with UTI (RRR = 0.00, p = 0.99) and SAM (RRR = 0.00, p = 0.99) had lower RRR of being moderately anemic.

The coefficient for living in urban areas (RRR = 0.85, p = 0.92) shows a lower RRR of being in this category as compared to other variables. Girls (RRR = 1.18, p=0.66) and children above two years of age (RRR = 1.98, p = 0.08) were insignificantly related to high RRR. The diagnosis of pneumonia (RRR = 5.61, P < 0.05) and other diagnoses (RRR = 5.87, p < 0.02) had significantly higher RRRs of being mildly anemic compared to other diagnoses at admission.

**Table 4:** The multinomial logistic regression of the hospitalized children on the grading of anemia severity (n=252) Abbreviations: n: positive number; RRR: relative risk reduction; AWD: acute watery diarrhea; UTI: urinary tract infection; SAM: severe acute malnutrition

Predictor Variables	Grade of Anemia								
	Mild			Moderate			Severe		
	n	RRR	95% CI	n	RRR	95% CI	n	RRR	95% CI
Sex									
* Males*	15	1.00	(base)	66	1.00	(base)	65	1.00	(base)
* Females*	8	1.18	0.57-2.45	47	0.57	0.21-1.57	51	0.89	0.51-1.54
Age									
* 6 – 24*	13	1.00	(base)	75	1.00	(base)	66	1.00	(base)
* 25 – 59*	10	1.98	0.93-4.19	38	1.46	0.53-3.98	50	0.75	0.43-1.32
Diagnosis at admission									
* Malaria*	7	1.00	(base)	64	1.00	(base)	87	1.00	(base)
* Pneumonia*	7	5.61	2.12-14.87	16	7.07	2.05-24.35	14	1.44	0.65-3.21
* Other diagnoses*	3	5.87	1.94-17.82	7	4.72	0.99-22.33	8	1.21	0.42-3.54
* Septicemia*	1	4.41	0.66-29.51	12	4.58	0.40-52.21	3	4.93	1.31-18.55
* AWD*	3	0.00	0-.	3	0.00	0-.	0	27.76	0-.
* UTI*	0	12.40	1.02-15.30	4	0.00	0-.	1	5.12	0.55-47.53
* SAM*	0	1.55	0.15-16.17	3	0.00	0-.	3	1.45	0.28-7.52
* Gastroenteritis*	2	85.00	0-.	4	0.00	0-.	0	44.65	0-.
Residence									
* Rural*	12	1.00	(base)	57	1.00	(base)	63	1.00	(base)
* Urban*	11	0.85	0.41-1.79	56	0.92	0.35-2.41	53	1.09	0.63-1.90

Classification of Anemia by Cell Size (MCV)

We also performed a multinomial logistic regression analysis to assess the predictors of the morphological types of anemia among children (Table 5). Predictor variables in the model included sex, age, and WBC counts. Microcytic anemia was found in 33.18 percent (n=70) of anemic children. Children with leucopenia (RRR = 0.85, p = 0.73) had lower RRR of having microcytic anemia, while female gender, age above two years, and leucocytosis were related to high RRR. Normocytic anemia was reported in 50.71 percent (n=107) of all anemic patients. Girls (RRR = 0.68, p=0.32) were associated with lower RRR of having normocytic anemia. In the macrocytic anemia category, age above two years (RRR = 0.67, p = 0.83) indicates a lower RRR of being in this category as compared to other variables.

**Table 5:** The multinomial logistic regression of the hospitalized children on the classification of anemia by cell size (MCV) (n=211) Abbreviations: n, positive number; RRR: relative risk reduction; AWD: acute watery diarrhea; UTI: urinary tract infection; SAM: severe acute malnutrition; MCV: mean corpuscular volume

Predictor Variables	MCV								
	Microcytic			Normocytic			Macrocytic		
	n	RRR	95% CI	n	RRR	95% CI	n	RRR	95% CI
Sex									
* Males*	37	1.00	(base)	55	1.00	(base)	34	1.00	(base)
* Females*	33	1.23	0.63-2.37	52	0.68	0.27-1.72	23	0.91	0.54-1.53
Age									
* 6 – 24 months*	42	1.00	(base)	60	1.00	(base)	27	1.00	(base)
* 25 – 59 months*	28	1.37	0.71-2.66	47	1.02	0.41-2.50	30	0.67	0.39-1.14
WBC Counts									
* Normal *	42	1.00	(base)	66	1.00	(base)	20	1.00	(base)
* Leucopenia *	10	0.85	0.41-1.79	27	1.02	0.35-2.41	17	1.09	0.63-1.90
* Leucocytosis*	18	1.81	0.18-18.54	14	1.76	0.93-2.71	20	1.55	0.72-3.41

## Discussion

We analyzed a cohort of hospitalized children under five years of age for the prevalence of anemia and the degree of severity and morphological characteristics of red blood cells (RBCs). This study found 83.17 percent of hospitalized children under five years of age had some level of anemia (Hb < 11g/dl), indicating that the prevalence of anemia is a severe public health problem in southern Tanzania. This prevalence was higher than that reported by studies elsewhere [11, 15-19]. Various reasons may have accounted for these differences. First, the study population consisted entirely of sick children who had been admitted to hospital. Some of the children had probably acquired anemia prior to admission or they had developed anemia while being attended at the hospital for other diseases, adding to this high prevalence. Second, the study area is among the regions that have a large number of poor people with unstable socio-economic status. Third, this is an area where the availability of health personnel and health-care facilities is scarce. Fourth, the study area is among the regions heavily affected by malaria and parasitic infestations, which are important risk factors for anemia.  

WHO classifies the severity of anemia into three grades, mild, moderate, and severe [13], based on hemoglobin concentration and gender. The reported prevalence of severe anemia (46.03 percent) in this study is relatively higher than that observed in one study (27.00 percent) in north-western Tanzania [11]. The identification of severe anemia in this age group is important because of the anticipated risks of morbidity and mortality and derailed milestones. The higher prevalence of malaria reported in this study may be one of the significant contributory factors for the high prevalence of severe anemia. Similar to our findings, several studies have also reported malaria as an important cause of severe anemia [2, 20] with various mechanisms of erythrocyte destruction by malaria leading to anemia having previously been described [21]. Generally, findings from our study and those reported in the literature [11,16] reveal a higher prevalence in hospital-based cohorts than in community cohorts. 

We found a slightly higher prevalence of anemia among boy cohorts than in among girl cohorts (84.88 percent vs 80.92 percent). Furthermore, findings showed a slightly higher prevalence of anemia among children below two years of age than in those above two years of age (86.03 percent vs 79.03 percent). This pattern was observed in all grades of anemia. The trend of the decreasing prevalence of anemia with age was also reported elsewhere [4,11,22] and may be attributed to increasing nutritional needs as the child grows and iron deficiency because of a complex of factors including worm infestations. On the other hand, differences in the prevalence of anemia in boys and girls may be due to, although not discussed in this study, the presence of SCD or thalassemia, which are diseases that normally affect more boys than girls. These differences in anemia prevalence in relation to gender and age are consistent to those reported elsewhere [9-11,15,22-24]. However, our findings did not show differences in the mean hemoglobin levels of boys and girls.

Previous studies have shown that the reduced renal production of erythropoietin because of renal failure was associated with anemia [25]. Because of limited data, this study could not show the relationship between renal dysfunction and the development of anemia. Our study also revealed that respiratory tract infections and AWD were associated with higher risks of developing anemia despite the latter accounting for only a small proportion of anemic children in this cohort. Furthermore, we found a large proportion of anemia cases were associated with malaria and respiratory tract infections. The findings support the fact that malarial infection contributes significantly to anemia in children under five. The infections are potentially preventable and treatable.

MCV is a way of classifying anemia based on RBC size [26]. In our study, normocytic anemia was the most prevalent type of morphological anemia in both sexes, accounting for 51 percent, which corroborates with findings from previous studies [7-8,27]. Macrocytic anemia was the least frequently observed subtype (16.11 percent) followed by microcytic anemia. In most cases, microcytic anemia results from an iron deficiency, while macrocytosis commonly results from megaloblastic anemia because of either a vitamin B12 or a folate deficiency. However, due to the nature of this study and limited data, the study failed to predict any underlying pathogeneses related to the observed microcytosis and macrocytosis.  

Leucopaenia resulting from absolute neutropenia, thrombocytopenia, and macrocytosis are suggestive features of the myelodysplastic syndrome [28]. This study found that about 29.82 percent of anemic children with macrocytosis also had leucopenia, however, because of the design of the study and limited data, we could not report platelet counts, leaving the WBC and MCV findings largely inconclusive. It is, as a result, not possible to evaluate the presence of the myelodysplastic syndrome in this group of children. Future studies should consider resolving the problem.

Our study has several limitations. We performed retrospective analyses based on a single available set of complete blood counts instead of repeated measurements. In this case, it was not possible to accurately differentiate between an acute and a chronic disease although cell volumes could, to some extent, differentiate between the two. There was also an unavailability of data that would otherwise aid in identifying the etiologies of anemia in this cohort. They included unavailable platelets counts, B12 levels, folate levels, SCD, thalassemia, glucose-6-phosphate dehydrogenase (G6PD) deficiencies, and bone marrow biopsies. Being a hospital-based study, our findings cannot be generalized to the general under-five population because the study had a biased sample of only sick, hospitalized children in southern Tanzania. Other limitations include the small sample size and the use of secondary data from a hospital. The study also could not ascertain the causal relationship among various factors on anemia. Because of the lack of local standards for the definition of anemia, we relied on the recommended WHO criteria to define and classify it. Furthermore, our study was limited by poor and unstandardized hospital documentation, which to a large extent, resulted from a lack of important data.  The unavailable data included a lack of stool analysis reports that could help evaluate the extent of worm infestation as an important cause of anemia in this cohort.

## Conclusions

The prevalence of anemia in hospitalized children under five years of age in southern Tanzania is high, with a significant proportion of cases of severe anemia. Malaria parasitemia is the most common disease associated with anemia. Our study underscores the relevance of anemia in hospitalized children and contributes to an understanding of its possible causes. We recommend routine screening for anemia during the admission of all children under the age of five years and thorough investigations to enable individualized treatments based on physical and laboratory findings.
